# Molecular mechanisms of garlic-derived allyl sulfides in the inhibition of skin cancer progression

**DOI:** 10.1111/j.1749-6632.2012.06743.x

**Published:** 2012-10-10

**Authors:** Hsiao-Chi Wang, Jung Pao, Shuw-Yuan Lin, Lee-Yan Sheen

**Affiliations:** 1 Institute of Food Science and Technology, National Taiwan UniversityTaipei, Taiwan, ROC; 2 Department of Cosmetics Applications and Management, Cardinal Tien College of Healthcare and ManagementNew Taipei, Taiwan, ROC; 3 Department of Nutrition, Hung-Kuang UniversitySha Lu, Taichung, Taiwan, ROC

**Keywords:** allyl sulfides, apoptosis, ER stress, garlic, ROS, skin cancer

## Abstract

Skin cancer is a serious concern whose incidence is increasing at an alarming rate. Allyl sulfides—i.e., sulfur metabolites in garlic oil—have been demonstrated to have anticancer activity against several cancer types, although the mechanisms underlying these effects remain enigmatic. Our previous study showed that diallyl trisulfide (DATS) is more potent than mono- and disulfides against skin cancer. DATS inhibits cell growth of human melanoma A375 cells and basal cell carcinoma (BCC) cells by increasing the levels of intracellular reactive oxygen species (ROS) and DNA damage and by inducing G2/M arrest, endoplasmic reticulum (ER) stress, and mitochondria-mediated apoptosis, including the caspase-dependent and -independent pathways. This short review focuses on the molecular mechanisms of garlic-derived allyl sulfides on skin cancer prevention.

## Introduction

Skin cancer, the most prevalent cancer worldwide, can be broadly divided into melanoma and nonmelanoma skin cancer (which includes basal and squamous cell carcinoma), depending on the cell type. Epidemiological studies show that the incidence of skin cancer has increased at an alarming rate in previous decades not only in Western countries but also in Asia.^1^–^3^ The World Health Organization claims that one in three cancer cases are skin related, which stresses the global importance of skin cancer prevention. Basal cell carcinoma (BCC) is the most common type of keratinocyte tumor, exhibiting slow growth rate and spreading rarely.^4^ In contrast, due to its rapid metastasis and chemotherapy resistance, melanoma is the least common form but most lethal malignancy derived from melanocytes.^5^ Evidence strongly suggests that excessive ultraviolet radiation (UVR) exposure, ozone depletion, genetic and dietary factors, and lifestyle are associated with the development of skin cancer.^6^,^7^ It is well known that skin cancer results from cocarcinogenic effects of different events, such as UVR-induced DNA damage, oxidative stress, inflammation, and immune suppression.^6^,^8^ Therefore, many natural dietary agents have received considerable attention because of their biological effects, such as antioxidant, anti-inflammatory, and anticarcinogenic functions.^9^,^10^

Epidemiological studies have suggested that the traditional Mediterranean diet containing high levels of antioxidants and phytochemicals has been associated with decreased incidence of skin cancer.^11^ Garlic (*Allium sativam L.*) is widely used in traditional herbal remedies and alternative medicine. The National Cancer Institute (NCI) set garlic on the top of a vegetable pyramid, representing potency in cancer prevention.^12^ The anticarcinogenic effect of garlic is attributed to the presence of organosulfur compounds such as allicin, allyl sulfides, ajoene, and *S*-allyl cysteine (SAC).^13^ Fresh garlic cloves contain 0.2–0.5% garlic oil in the steam-distilled materials. Allyl sulfides, including diallyl sulfide (DAS), diallyl disulfide (DADS), diallyl trisulfide (DATS), and other allyl polysulfides are the most abundant compounds in garlic oil, accounting for nearly 94% of the total amount.^14^ The proportion of allyl sulfides in garlic oil consists of approximately 4.7–8% DAS, 21.9–40% DADS, and 39–41.5% DATS, which depends on the extraction conditions.^15^,^16^ Much evidence suggests that these allyl sulfides suppress the growth of multiple cancer types in both *in vitro* and *in vivo* models.^17^,^18^ Here, we succinctly review the current literature pertaining to anticancer properties of garlic oil and allyl sulfides against skin cancer, with special emphasis on the potential mechanisms.

## Inhibitory action of garlic-derived allyl sulfides on chemical carcinogen-induced skin cancer in mice

Skin carcinogenesis is a multistage process involved in the alteration of the signaling molecules regulating cell proliferation, differentiation, and death activated by UV radiation or chemical carcinogens. These signaling molecules contain various transcription factors (e.g., p53, p21, activator protein-1 (AP-1)), cell cycle proteins (e.g., cyclins, cyclin-dependent kinases), antiapoptotic proteins (e.g., Bcl-2, Bcl-xl), proapoptotic proteins (e.g., Bax, caspases), inflammatory enzymes (e.g., cycloxygenase-2 (COX-2)), numerous protein kinases (e.g., c-jun *N*-terminal kinase (JNK), Akt protein kinase), cell adhesion molecules, and growth factor signaling pathways. Therefore, agents that might prevent precancerous lesions caused by environmental carcinogens or that possess UV-blocking, antioxidant, antimutagenic, and anti-inflammatory properties may be useful against skin cancer.^19^,^20^

The chemical carcinogen–induced two-stage skin tumor is a well-established animal model to study mechanisms of epithelial carcinogenesis. The initiation stage involving irreversible mutation in H-ras proto-oncogene is accomplished by the application of a carcinogen, such as 7, 12-dimethyl benz(a)anthracene (DMBA). Subsequently, carcinogen-initiated skin is induced by repeated treatment with a tumor-promoting agent, such as 12-*O*-tetradecanoylphorbol-13-acetate (TPA) or phorbol myristate acetate (PMA), which causes the formation of benign tumors or papilloma.^20^,^21^ In this model, while the early stage of promotion is reversible, late-stage promotion and progression exhibit the irreversible phases of the tumorigenesis process.^21^ Belman was the pioneer to reveal chemopreventive effects of garlic oil against skin tumorigenesis initiated by DMBA and promoted by PMA.^22^ Published results documenting efficacy of garlic-derived allyl sulfides against chemical carcinogen-induced skin cancer in experimental rodents are summarized in Table 1. From these studies, it is clear that topical applications of garlic oil or allyl sulfides inhibit skin papilloma formation, reduce tumor incidence, and increase the survival rate in mice.^23^–^26^ Several mechanistic studies have been proposed to explain the antitumorgenesis effect of DAS, including modulating carcinogen metabolism, inhibiting carcinogen-induced DNA damage, increasing cellular defenses system, and leading to apoptosis in carcinogen-induced skin tumor.^27^–^33^ Kalra *et al.* showed that DAS suppresses DMBA-induced skin tumors through induction of apoptosis via modulation of ras-induced phosphatidylinositol 3-kinase (PI3K)/Akt, mitogen-activated protein kinase (MAPKs), and p53-mediated signaling pathways.^30^ Among the garlic-derived allyl compounds, DATS was more potent than DAS and DADS to suppress TPA-induced COX-2 expression. The antitumor-promoting effect of DATS on TPA-induced COX-2 and AP-1 expression is involved in modulation of JNK or Akt signaling on mouse skin carcinogenesis.^34^ Taken together, the prevention of carcinogenic progression by allyl sulfides has been attributed to its strong antioxidant, anti-inflammatory, and antiproliferation properties. Allyl sulfides provide a multiprong beneficial approach for targeting multiple signaling pathways in skin cancer prevention.

**Table 1:** Topical application of garlic oil and allyl sulfides protect against chemical-induced skin carcinogenesis in mice

Agents	Experimental design	Dose	Findings	References
Garlic oil	DMBA + PMA female Ha/ICR mice	10, 100 μg, 1mg	↓ Number of tumors per surviving mouse	Belman^22^
	177 days		↓ Percent living mice with tumors	
	Benzo[a]pyrene (BP) + croton oil female Swiss albino mice	10% garlic oil/0.1 mL acetone	↓ Number of tumor-bearing mice	Sadhana *et al.*^23^
			↓ Number of tumors per effective mouse	
	16 weeks			
	DMBA + PMA SENCAR mice	2 mg garlic oil/0.2 mL acetone	↓ Number of papillomas/mouse	Perchellet *et al.*^24^
			↓Percent of mice with papillomas	
	18 weeks			
DAS	DMBA + benzoyl peroxide (BPO) SENCAR mice	20 μM/0.2 mL acetone/mouse	↓ Tumor incidence	Athar *et al.*^25^
			↓ Number of papillomas/mouse	
			↓ Number of carcinomas/mouse	
	51 weeks			
	DMBA female Swiss albino mice	250 μg /0.1 mL acetone	↓ Tumor incidence	Singh *et al.*^31^
			↓ Average number of tumors/mouse	Arora *et al.*^32^,^33^,^35^
	28 weeks			
			↑ Apoptosis	
			↑ Wild-type p53 expression	
			↓ Mutant p53 expression	
			↓ DMBA-induced H-ras mRNA level and p21/ras expression	
	DMBA female Swiss albino mice	2.5, 5, 10 mg/kg BW	↓ DMBA-induced DNA strand breaks	Nigam and Shukla^29^
	96 hours		↓DMBA-induced overexpression of ras oncoprotein	Kalra *et al.*^30^
			↓ Ras-induced PI3K/Akt and p38MAPK pathway	
			↑ p53-mediated apoptotic pathway	
DADS	DMBA + TPA SENCAR mice	1 mg/0.1 mL acetone	↓ Number of papillomas/mouse	Dwivedi *et al.*^26^
DAS			↑ Survival rate	
	22 weeks			
DATS	DMBA + TPA female ICR mice	5, 25 μg/0.2 mL of acetone/DMSO	↓ Incidence and multiplicity of papillomas	Shrotriya *et al.*^34^
	20 weeks		↓ TPA-induced AP-1 activation and COX-2 expression via modulation of the JNK or Akt signaling pathway	

The arrows indicate the increase (↑) or decrease (↓).

ICR, Imprinting Control Region; DMSO, dimethylsulfoxide.

## Mechanistic studies of growth inhibition of allyl sulfides in skin cancer cells

Research has focused on the anticancer effect of allyl sulfides in culture and *in vivo* models, including prostate, lung, and colon cancers.^18^ Chemoprevention of skin cancer by garlic organosulfur has recently received increased attention.^30^,^35^,^36^ Extensive studies to elucidate the mechanism of DATS-induced cell cycle arrest and apoptosis using human melanoma A375 cells and BCC cells as a model have been done in our lab.^37^,^38^ A number of studies have indicated that the number of sulfur atoms on allyl sulfides determines their efficacy and biological activity, such as anticancer and anti-inflammatory effects.^39^ The ability of allyl sulfides to suppress the growth of cancer cells tightly correlates with the length of the sulfur chain.^40^ In line with previous reports, we revealed that DATS (25 μM) was more effective than DADS and DAS in decreasing cell viability of A375 and BCC cells. Moreover, DATS inhibited cell growth of A375 and BCC cells via activation of multiple target pathways.^37^,^38^ The chemical properties and mechanisms determining the anticancer action of garlic-derived allyl sulfides have attracted recent scientific interest.^40^ Studies have shown that the antiproliferative effects of garlic-derived allyl sulfides are associated with their conversion to sulfane sulfur in tumor cells and/or to controlling proliferative signals.^41^ For example, garlic organosulfur compounds bearing an *S*-allyl moiety can directly or indirectly target the redox-sensitive proteins at sulfhydryl sites, including cell-cycle checkpoint control proteins, apoptotic regulatory proteins, and transcription factors.^42^ Results from our findings and other studies are discussed in the following section to illustrate the mechanism of skin cancer prevention by garlic-derived allyl sulfides.

## Induction of ROS generation and DNA damage in skin cancer cells

Accumulating evidence indicates that human tumors frequently have defects in response to oxidative stress and DNA damage, compared with normal human cells. Hence, a DNA damage agent with less normal tissue toxicity is one of the newest cancer therapies.^43^ Studies have shown allyl disulfides can produce reactive oxygen species (ROS) directly by reactions relying upon the homolytic cleavage of disulfide bonds. Allyl sulfides can contribute to the generation of ROS, the depletion of glutathione (GSH), and the establishment of pro-oxidant conditions in cancer cells.^44^ The differences between disulfides and trisulfides in the capability of ROS production and inability of monosulfides to form ROS can explain their diverse toxicities. Many authors revealed that the cytotoxicity of different organosulfurs under similar concentration decreases in the following order DATS > DADS > DAS.^45^ Our previous study demonstrated that the dose-dependent cytotoxicity induced by DATS in A375 and BCC cells was associated with both an increase in the level of ROS and expression of DNA damage markers, including γ-H_2_AX, phospho-p53 (Ser 15), and p21.^37^ In addition, the pretreatment of antioxidant *N*-acetyl cysteine (NAC) suppressed ROS generation, DNA damage, and cytotoxicity in BCC cells caused by the treatment of DATS (100 μM).^38^ A considerable amount of research has indicated that DNA damage–induced p53 pathways (e.g., postmitotic cell death, cell cycle arrest) are involved in antiproliferative effect of chemotherapeutic agents.^46^ Our results suggest the possibility that ROS-mediated oxidative DNA damage plays an important role in DATS-induced cell death.^37^

## Induction of cell cycle arrest at the G2/M phase

Recent studies have shown that allyl sulfides induce G2/M phase cell cycle arrest in several human cancer cells.^47^,^48^ The regulation of cell cycle progression in human cancer cells is connected with the modulation of allyl sulfides on checkpoint modulators, such as cyclin-dependent kinase 1 (Cdk1) and cell division cycle 25 C (Cdc25 C) phosphatase.^49^ Xiao *et al*. demonstrated that DATS induced G2/M arrest of human prostate cancer cells by ROS-mediated hyperphosphorylation of Cdc25 C was associated with increased Tyr15 phosphorylation of Cdk1 and inhibition of Cdk1/cyclinB1 kinase activity.^50^ It is known that Cdc25 C phosphatases and Wee 1 kinase play critical roles in maintaining G2 phase arrest through modulating the phosphorylation of Cdc2.^51^ In agreement with previous findings, our study revealed that DATS (25 μM) decreased the expression of Cdc25 C and Cdc2, and increased the levels of Wee 1 and cyclin B1 expression in a time-dependent manner, which resulted in accumulation of sub-G1 population in A375 and BCC cells. DNA damage and ROS generation was attributed to DATS-mediated G2/M arrest in these two types of skin cancer cells. Subsequently, DATS caused a temporary G2/M arrest, which was followed by an increasing percentage of polyploid DNA content and sub-G1 DNA content by the cell cycle analysis at different time points (0, 3, 6, 12, and 24 h).^37^ These results are in line with reports that cells without complete DNA repair can result in mitotic arrest, errors in chromosome segregation, and formation of aberrant polyploidy cells, subsequently leading to cell death.^46^ Our ongoing work also shows the concordance for the expressions of molecules involved in G2/M arrest induced by garlic oil. Collectively, these studies indicate that the induction of cell cycle arrest in cancer cells is a common cellular response to garlic-derived allyl sulfides.

## Induction of apoptosis through the mitochondria pathway

Evidence suggests that the suppression of cancer cell growth by garlic oil and allyl sulfides correlates with apoptosis induction.^18^,^52^ Milner *et al.* was the first to report DADS-induced apoptosis observed by DNA fragmentation and other morphological changes in human colon cancer cells.^53^ Most studies implicate involvement of disrupting the balance of the Bcl-2 family proteins in regulation of the allyl sulfides–mediated mitochondrial apoptosis pathway.^49^ Clinical observation of patients revealed that overexpression of antiapoptotic Bcl-2 protein enhances cell survival and contributes to the severity of aggressive skin tumors.^54^ A therapeutic trial from Tilli *et al.* found that topical application of ajoene onto tumors in 21 patients with nodular or superficial basal cell carcinoma for six months reduced tumor size in 17 cases, with a concomitant decrease in the expression of Bcl-2 protein in the tumor cells, as evaluated by immunohistochemical assays. Moreover, the results of *in vitro* study suggested that the antitumor effect of ajoene was associated with induced mitochondria-dependent apoptosis.^55^ The mitochondrial apoptosis response is associated with different phenomenon, including the disruption of mitochondrial membrane potential, an altered ratio of proapoptotic protein Bax and antiapoptotic protein Bcl-2, stimulation of the release of cytochrome *c* from the mitochondria into the cytosol, and the activation of apoptotic protease activating factor 1 (Apaf-1), caspase-9, caspase-3, and poly (ADP-ribose) polymerase (PARP).^56^ Studies have shown that Bcl-2 phosphorylation leads to reduced formation of Bax-Bcl-2 heterodimers and activation of the mitochondria-mediated intrinsic caspase cascade.^57^ Consistent with previous results, our study demonstrated that DATS (25 μM) induced apoptosis of A375 and BCC cells via the mitochondrial pathway. DATS decreased the antiapoptotic levels of Bcl-2 and Bcl-xl, increased the expression of Bax, and activated Bcl-2 phosphorylation in A375 and BCC cells, which correlated with loss of the mitochondrial membrane potential.^37^,^38^

It is well known that modulation of the p53-mediated pathway is one of the major mechanisms to induce cell death for dermatological treatments. However, melanoma cells have a low frequency of spontaneous apoptosis *in vivo* and are relatively resistant to the agents used in chemotherapy *in vitro*. Studies have reported that A375 and BCC cells exhibit wild-type p53 function, which is activated upon apoptosis by a variety of cellular stresses. The phosphorylation at serine 15 of p53 protein plays a role in responding to cellular DNA damage and the occurrence of p53-mediated apoptosis.^58^,^59^ Our earlier study suggested that DATS triggered DNA damage and, consequently, induced G2/M arrest and apoptosis through the p53 pathway.^37^,^38^ Arora *et al.* showed that induction of apoptosis and modulation of the tumor suppressor p53 as plausible mechanisms of the antiproliferative effect of allyl sulfide in DMBA-induced mouse skin tumors.^32^,^33^ Another study by Kalra *et al.* demonstrated that the antitumorigenesis effect of DAS is due to the upregulation of p53 that is linked to its downstream target molecule p21, and downregulation of ras oncoprotein, antiapoptotic proteins survivin, and Bcl-2.^30^ Therefore, these studies suggest that the p53 pathway may play an important role in allyl sulfide–induced apoptosis and cell death of skin cancer cells.

A recent study has shown that the activation of both caspase-dependent and -independent mitochondrial pathways leads to observable inhibition of cancer cells and results in a lower level of tumor resistance.^60^ Although much evidence indicates that allyl sulfides induce apoptosis of several human cancer cell types via the mitochondrial caspase-dependent pathway, little is known about their effect on the caspase-independent pathway. Among the caspase-independent molecules, apoptotic induced factor (AIF) and endonuclease G (Endo G) are directly involved in DNA fragmentation after nuclear translocation in caspase-independent cell death.^61^ Moreover, HtrA_2_/Omi induces both caspase-dependent and -independent pathways by decomposing members of the inhibitor of apoptosis proteins (IAPs) family and decreasing their serine protease activity.^62^ Studies from our laboratory have revealed that DATS upregulates the expression of cytosolic AIF, HtrA_2_/Omi, nuclear AIF, and Endo G in a dose-dependent manner, providing the novel finding that DATS induces both caspase-dependent and -independent apoptosis in skin cancer cells.^38^

## Endoplasmic reticulum stress-induced apoptosis

Growing evidence indicates that induction of DNA damage activates the unfolding protein response and endoplasmic reticulum (ER) stress-induced apoptosis.^46^ Upon exposure to ER stress, cytosolic Ca^2+^ levels are increased due to release of Ca^2+^ from internal stores. Subsequently, cytosolic Ca^2+^ mobilization causes mitochondrial membrane potential depolarization and apoptosis via the caspase cascade.^63^ Among the ER-associated apoptotic molecules, GRP78/BiP (glucose-regulated protein of 78 kDa), CHOP/GADD153, and caspase-4 are well-known biomarkers or proapoptotic factors that are closely associated with ER stress.^64^ Das *et al.* were first to reveal DATS-mediated cell death via ER stress induction in human glioblastoma cells; however, its mechanism of action remains uncharacterized.^65^ Sundaram *et al.* have shown that increasing intracellular free calcium is associated with the antiproliferative effect of DADS in human melanoma SK MEL-2 cells.^66^ We found that the induction of ER stress markers, CHOP/GADD153 and BiP/GRP78, is correlated with increased intracellular free Ca^2+^ levels, mitochondrial membrane depolarization, and activation of caspase-4 in BCC cells after the treatment of DATS (25 μM).^38^ Studies have demonstrated that ER stress activates the unfolding protein response and the ER-resident cysteine protease, initiating the caspase cascade and amplifying the proapoptotic signal by altering the balance between Bcl-2 and Bax, and inducing caspase-dependent and -independent pathways.^67^ Our data support the fact that activation of the ER stress pathway triggers apoptosis upon DATS treatment in A375 and BCC cells. In our current research program, studies are in progress to explore whether garlic oil (mixture of mono-, di-, and tri-allyl sulfides) has a similar molecular mechanism to that of DATS in inhibiting growth of human skin cancer cells.

## Inhibition of human melanoma metastasis *in vitro*

Epidemiological studies have identified that melanoma is a lethal type of skin cancer because of its relatively high probability to progress to metastasis and its resistance to chemotherapy.^68^ Therefore, it is imperative to develop efficacious preventive and therapeutic strategies for melanoma. Taylor *et al.* were the first to report the antimetastatic effect of garlic sulfur compounds. The result has shown that ajoene inhibits tumor cell growth *in vitro*, and strongly suppresses metastasis to lung in the B16/BL6 melanoma cell model in C57BL/6 mice.^69^ Our studies are in progress to demonstrate that DATS inhibits cell migration, adhesion, and invasion of A375 cells under noncytotoxic concentration as analyzed by wound healing assays and via a Matrigel invasion chamber system. The antimetastatic potency might be related to the decrease in the activity of matrix metalloproteinases (MMPs) induced by DATS, including MMP-2 and MMP-9. Similarly, a pervious study revealed that the antiadhesion effect of allyl sulfides involved in their sulfide group interacts with membrane lipids to modify the membrane fluidity or protein.^70^ These results provide new insight into the mechanisms of allyl sulfides on modulating the metastatic potency of human cancer cells. Our ongoing studies are exploring the mechanism for antimetastatic action of DATS on human melanoma cells.

**Figure 1:**
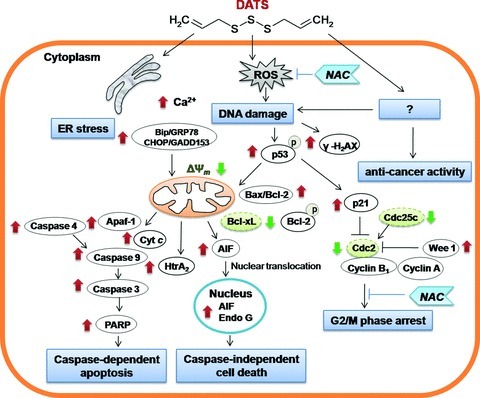
Proposed mechanisms for DATS-induced cell cycle arrest and apoptosis induction in skin cancer cells. The arrows indicate the expression changes in our results. The DATS treatment increases the ROS level and inflicts DNA damage, which is reflected by the increase of γ-H_2_AX and consequent activation of the p53/p21 and affects G2/M modulator, such as Wee l kinase, Cdc25C, and Cdc2. DATS also induces Ca^2+^ mobilization and ER stress-related molecules such as Bip/GRP78 and CHOP/GADD153, which trigger caspase-4 and caspase-9 activation before causing apoptosis. DATS induces caspase-dependent apoptosis by decreasing Bcl-xL expression, increasing the Bax/Bcl-2 ratio and Bcl-2 phosphorylation, and the subsequent loss of mitochondrial membrane potential. The release of mitochondrial proteins, such as Cyt *c*, AIF, HtrA_2_/Omi, and Endo G. Cytosolic Cyt *c,* in turn, activates the downstream effectors Apaf-1, caspase-9, caspase-3, and PARP cleavage. Alternatively, DATS-triggered caspase-independent cell death is facilitated by increased nuclear translocation of AIF and Endo G. Moreover, antioxidant NAC suppressed DATS-induced ROS generation, growth inhibition, G2/M arrest, and apoptosis of skin cancer cells. NAC, *N*-acetyl-l-cysteine; ROS, reactive oxygen species; ER, endoplasmic reticulum; Cyt c, cytochrome c; PARP, poly (ADP-ribose) polymerase; AIF, apoptotic-inducing factor; and Endo G, endonuclease G.

All of the above studies, carried out on cell culture and animal models, suggest that garlic oil and allyl sulfides are effective in imparting protection against skin cancer. The mechanisms of DATS-induced G2/M phase cell cycle arrest and apoptosis in skin cancer cells are summarized in Figure 1. Mechanisms underlying the skin cancer preventative effects of DATS are not completely understood, but known cellular responses to garlic-derived allyl sulfides include elevation of ROS and DNA damage, alteration of mitogenic and survival singling, and induction of G2/M arrest and apoptosis. Interestingly, we found that antioxidant NAC suppressed DATS-induced ROS production and growth inhibition. Although NAC nearly abolished DATS-induced DNA damage, it only partially blocked the effect of DATS on growth suppression, indicating that the ROS-independent pathway is involved in DATS-induced cell death. Moreover, our study has shown that DATS-mediated G2/M arrest and apoptosis appeared to be selective for cancer cells, since normal HaCaT cells were resistant to growth inhibition by DATS.^37^ This finding is consistent with previous studies in several cell and animal models.^50^ In our current research program, proteomic and genomic studies are in progress to further elucidate the different molecular events involved in skin cancer prevention by garlic oil and DATS in an animal model.

## Conclusions and future prospects

Accumulating experimental data indicate that garlic-derived allyl sulfides possess an anticancer effect in several organs, including the skin. The primary discussion in this review has centered around the chemopreventive skin cancer effect and the mechanism of allyl sulfides in a chemical carcinogen-induced mouse skin cancer and cell line model. However, the photopreventive effect of garlic-derived allyl sulfides is lacking. Studies have shown that UV-induced photocarcinogenesis in the hairless mouse is widely accepted as a reliable preclinical animal model for the evaluation of chemoprevention agents.^71^ Therefore, future studies should investigate the effect and mechanisms of allyl sulfides in preventing photocarcinogenesis. Moreover, the pharmacokinetics, bioavailability, and clinical investigations of DATS should be important for future recommendations for the practical application of garlic in the chemoprevention of skin cancer.

## References

[1] Sng J (2009). Skin cancer trends among Asians living in Singapore from 1968 to 2006. J. Am. Acad. Dermatol.

[2] Liu Y (2010). A content analysis of news coverage of skin cancer in China newspapers. Health Commun.

[3] Ohtsuka H, Nagamatsu S (2005). Changing trends in the number of deaths from nonmelanoma skin cancer in Japan, 1955–2000. Dermatology.

[4] Kim RH, Armstrong AW (2012). Nonmelanoma skin cancer. Dermatol. Clin.

[5] Nikolaou VA (2012). Melanoma: new insights and new therapies. J. Invest. Dermatol.

[6] Pfeifer GP, Besaratinia A (2012). UV wavelength-dependent DNA damage and human non-melanoma and melanoma skin cancer. Photochem. Photobiol. Sci.

[7] van der Pols JC (2010). Food intake and risk of basal cell carcinoma in an 11-year prospective study of Australian adults. Eur. J. Clin. Nutr.

[8] Halliday GM (2010). Common links among the pathways leading to UV-induced immunosuppression. J. Invest. Dermatol.

[9] Katiyar SK (2007). UV-induced immune suppression and photocarcinogenesis: chemoprevention by dietary botanical agents. Cancer Lett.

[10] Nichols JA, Katiyar SK (2010). Skin photoprotection by natural polyphenols: anti-inflammatory, antioxidant and DNA repair mechanisms. Arch. Dermatol. Res.

[11] Vinceti M (2005). A population-based case–control study of diet and melanoma risk in northern Italy. Public Health Nutr.

[12] Steinmetz KA, Potter JD (1996). Vegetables, fruit, and cancer prevention: a review. J. Am.Diet. Assoc.

[13] Block E (1985). The chemistry of garlic and onions. Sci. Am.

[14] Calvo-Gomez O, Morales-Lopez J, Lopez MG (2004). Solid-phase microextraction-gas chromatographic-mass spectrometric analysis of garlic oil obtained by hydrodistillation. J. Chromatogr. A.

[15] Sheen LY, Lin SY, Tsai S-J (1991). Preparation of spray-dried microcapsules with various amounts of basil, garlic and ginger essential oils and changes in oil signal transduction pathways. Biochem. Pharmacol.

[16] Lawson LD, Wang ZJ, Hughes BG (1991). Identification and HPLC quantitation of the sulfides and dialk(en)yl thiosulfinates in commercial garlic products. Planta Med.

[17] Powolny AA, Singh SV (2008). Multitargeted prevention and therapy of cancer by diallyl trisulfide and related Allium vegetable-derived organosulfur compounds. Cancer Lett.

[18] Raghu R (2012). Recent research progress on garlic as a potential anticarcinogenic agent against major digestive cancers. J. Trad. Complement. Med.

[19] Nishigori C (2006). Cellular aspects of photocarcinogenesis. Photochem. Photobiol. Sci.

[20] Abel EL (2009). Multi-stage chemical carcinogenesis in mouse skin: fundamentals and applications. Nat. Protoc.

[21] Mukhtar H, Agarwal R (1996). Skin cancer chemoprevention. J. Investig. Dermatol. Symp. Proc.

[22] Belman S (1983). Onion and garlic oils inhibit tumor promotion. Carcinogenesis.

[23] Sadhana AS (1988). Inhibitory action of garlic oil on the initiation of benzo[a]pyrene-induced skin carcinogenesis in mice. Cancer Lett.

[24] Perchellet JP, Perchellet EM, Belman S (1990). Inhibition of DMBA-induced mouse skin tumorigenesis by garlic oil and inhibition of two tumor-promotion stages by garlic and onion oils. Nutr. Cancer.

[25] Athar M (1990). Inhibition of benzoyl peroxide-mediated tumor promotion in 7,12-dimethylbenz(a) anthracene-initiated skin of Sencar mice by antioxidants nordihydroguaiaretic acid and diallyl sulfide. J. Invest. Dermatol.

[26] Dwivedi C (1992). Chemoprevention of chemically induced skin tumor development by diallyl sulfide and diallyl disulfide. Pharm. Res.

[27] Arora A, Kalra N, Shukla Y (2006). Regulation of p21/ras protein expression by diallyl sulfide in DMBA induced neoplastic changes in mouse skin. Cancer Lett.

[28] Das I, Saha T (2009). Effect of garlic on lipid peroxidation and antioxidation enzymes in DMBA-induced skin carcinoma. Nutrition.

[29] Nigam N, Shukla Y (2007). Preventive effects of diallyl sulfide on 7,12-dimethylbenz[a]anthracene induced DNA alkylation damage in mouse skin. Mol. Nutr. Food Res.

[30] Kalra N, Arora A, Shukla Y (2006). Involvement of multiple signaling pathways in diallyl sulfide mediated apoptosis in mouse skin tumors. Asian Pac. J. Cancer Prev.

[31] Singh A, Shukla Y (1998). Antitumor activity of diallyl sulfide in two-stage mouse skin model of carcinogenesis. Biomed. Environ. Sci.

[32] Arora A, Shukla Y (2002). Induction of apoptosis by diallyl sulfide in DMBA-induced mouse skin tumors. Nutr. Cancer.

[33] Arora A, Siddiqui IA, Shukla Y (2004). Modulation of p53 in 7,12-dimethylbenz[a]anthracene-induced skin tumors by diallyl sulfide in Swiss albino mice. Mol. Cancer Ther.

[34] Shrotriya S (2010). Diallyl trisulfide inhibits phorbol ester-induced tumor promotion, activation of AP-1, and expression of COX-2 in mouse skin by blocking JNK and Akt signaling. Cancer Res.

[35] Arora A, Kalra N, Shukla Y (2006). Regulation of p21/ras protein expression by diallyl sulfide in DMBA induced neoplastic changes in mouse skin. Cancer Lett.

[36] Nishikawa T (2002). Inhibition by ajoene of skin-tumor promotion in mice. Biosci. Biotechnol. Biochem.

[37] Wang HC (2010). Allyl sulfides inhibit cell growth of skin cancer cells through induction of DNA damage mediated G2/M arrest and apoptosis. J. Agric. Food Chem.

[38] Wang HC (2012). Diallyl trisulfide induces apoptosis of human basal cell carcinoma cells via endoplasmic reticulum stress and the mitochondrial pathway. Nutr. Cancer.

[39] Munchberg U (2007). Polysulfides as biologically active ingredients of garlic. Org. Biomol. Chem.

[40] Cerella C (2011). Chemical properties and mechanisms determining the anti-cancer action of garlic-derived organic sulfur compounds. Anticancer Agents Med. Chem.

[41] Pinto JT, Krasnikov BF, Cooper AJ (2006). Redox-sensitive proteins are potential targets of garlic-derived mercaptocysteine derivatives. J. Nutr.

[42] Xiao D (2003). Induction of apoptosis by the garlic-derived compound S-allylmercaptocysteine (SAMC) is associated with microtubule depolymerization and c-Jun NH(2)-terminal kinase 1 activation. Cancer Res.

[43] Powell SN, Bindra RS (2009). Targeting the DNA damage response for cancer therapy. DNA Repair.

[44] Filomeni G, Rotilio G, Ciriolo MR (2008). Molecular transduction mechanisms of the redox network underlying the antiproliferative effects of allyl compounds from garlic. J. Nutr.

[45] Ng KT (2012). A garlic derivative, S-allylcysteine (SAC), suppresses proliferation and metastasis of hepatocellular carcinoma. PLoS One.

[46] Varmark H (2009). DNA damage-induced cell death is enhanced by progression through mitosis. Cell Cycle.

[47] Wu CC (2004). Differential effects of allyl sulfides from garlic essential oil on cell cycle regulation in human liver tumor cells. Food Chem. Toxicol.

[48] Herman-Antosiewicz A (2010). Diallyl trisulfide-induced G2/M phase cell cycle arrest in DU145 cells is associated with delayed nuclear translocation of cyclin-dependent kinase 1. Pharm. Res.

[49] Iciek M, Kwiecien I, Wlodek L (2009). Biological properties of garlic and garlic-derived organosulfur compounds. Environ. Mol. Mutagen.

[50] Xiao D (2005). Diallyl trisulfide-induced G(2)-M phase cell cycle arrest in human prostate cancer cells is caused by reactive oxygen species-dependent destruction and hyperphosphorylation of Cdc 25 C. Oncogene.

[51] Stanford JS, Ruderman JV (2005). Changes in regulatory phosphorylation of Cdc25C Ser287 and Wee1 Ser549 during normal cell cycle progression and checkpoint arrests. Mol. Biol. Cell.

[52] Antony ML, Singh SV (2011). Molecular mechanisms and targets of cancer chemoprevention by garlic-derived bioactive compound diallyl trisulfide. Indian J. Exp. Biol.

[53] Sundaram SG, Milner JA (1996). Diallyl disulfide induces apoptosis of human colon tumor cells. Carcinogenesis.

[54] Correa Mde P (2009). Markers expression of cell proliferation and apoptosis in basal cell carcinoma. An. Bras. Dermatol.

[55] Tilli CM (2003). The garlic-derived organosulfur component ajoene decreases basal cell carcinoma tumor size by inducing apoptosis. Arch. Dermatol. Res.

[56] Li P (1997). Cytochrome c and dATP-dependent formation of Apaf-1/caspase-9 complex initiates an apoptotic protease cascade. Cell.

[57] Haldar S, Jena N, Croce CM (1995). Inactivation of Bcl-2 by phosphorylation. Proc. Natl. Acad. Sci. USA.

[58] Vogelstein B, Lane D, Levine AJ (2000). Surfing the p53 network. Nature.

[59] Koblish HK (2006). Benzodiazepinedione inhibitors of the Hdm2:p53 complex suppress human tumor cell proliferation in vitro and sensitize tumors to doxorubicin in vivo. Mol. Cancer Ther.

[60] Constantinou C, Papas KA, Constantinou AI (2009). Caspase-independent pathways of programmed cell death: the unraveling of new targets of cancer therapy. Curr. Cancer Drug. Targets.

[61] Widlak P, Garrard WT (2005). Discovery, regulation, and action of the major apoptotic nucleases DFF40/CAD and endonuclease G. J. Cell Biochem.

[62] Verhagen AM (2002). HtrA(2) promotes cell death through its serine protease activity and its ability to antagonize inhibitor of apoptosis proteins. J. Biol. Chem.

[63] Puzianowska-Kuznicka M, Kuznicki J (2009). The ER and ageing II: calcium homeostasis. Ageing Res. Rev.

[64] Schroder M (2008). Endoplasmic reticulum stress responses. Cell Mol. Life Sci.

[65] Das A, Banik NL, Ray SK (2007). Garlic compounds generate reactive oxygen species leading to activation of stress kinases and cysteine proteases for apoptosis in human glioblastoma T98G and U87MG cells. Cancer.

[66] Sundaram SG, Milner JA (1996). Diallyl disulfide inhibits the proliferation of human tumor cells in culture. Biochim. Biophys. Acta.

[67] He P (2010). Photoactivation of 9-hydroxyph eophorbide alpha triggers apoptosis through the reactive oxygen species-mediated mitochondrial pathway and endoplasmic reticulum stress in AMC-HN-3 laryngeal cancer cells. Int. J. Oncol.

[68] Cummins DL (2006). Cutaneous malignant melanoma. Mayo. Clin. Proc.

[69] Taylor P (2006). Ajoene inhibits both primary tumor growth and metastasis of B16/BL6 melanoma cells in C57BL/6 mice. Cancer Lett.

[70] Knowles LM, Milner JA (2000). Allyl sulfides modify cell growth. Drug. Metabol. Drug. Interact.

[71] Bowden GT (2004). Prevention of non-melanoma skin cancer by targeting ultraviolet-B-light signalling. Nat. Rev. Cancer.

